# Coating Formulations Based on Carbon Black: An Alternative to Develop Environmentally Friendly Conductive Cellulose Paper

**DOI:** 10.3390/ma18122708

**Published:** 2025-06-09

**Authors:** Adriana Millan, Anny Morales, Richard A. Venditti, Joel J. Pawlak

**Affiliations:** Department of Forest Biomaterials, College of Natural Resources, North Carolina State University, Raleigh, NC 27695-8005, USAacmorale@ncsu.edu (A.M.); richardv@ncsu.edu (R.A.V.)

**Keywords:** cellulose paper, functional coating, conductive coating, carbon black electrode

## Abstract

The current economic growth and increasing needs of society have led to developing processes that harm our environment and have severe long-term consequences. For this reason, different attempts have been made to mitigate these effects by substituting conventional toxic materials with environmentally friendly ones. Industry sectors related to energy storage, printed electronics, and wearable technology are moving towards applying sustainable strategies. Renewable biopolymers such as cellulose and its derivatives, as well as carbon-based alternatives, which include carbon nanotubes (CNTs), single-wall carbon nanotubes (SWCNTs), graphite, graphene, and carbon black (CB), are leading the advances in this field. The present research aimed to develop conductive cellulose paper using environmentally friendly components compatible with the paper recycling process. Coating formulations based on carbon black were proposed using three different types of binders: polytetrafluoroethylene (PTFE), latex (styrene butadiene), and sodium carboxymethyl cellulose (CMC). The formulation, composition, and preparation were studied, and they were related to the coating’s electrical resistance and integrity. This last parameter was determined through a new method described in this research, implementing a mechanical/optical technique to measure the coating’s durability. The formulation with the best performance in terms of electrical resistance (0.29 kΩ), integrity, and non-toxicity was obtained using sodium carboxymethyl cellulose (CMC) as a binder and dispersant.

## 1. Introduction

Flexible energy-storage devices have recently attracted interest from academia and industry due to their wide applications, including solar arrays, supercapacitors, flexible batteries, wearable technology, microelectronics, and so on [1,2]. Attempts to develop power sources matching the necessary properties, such as light weight, flexibility, and shape, have considerably increased [1,3,4] since the classic assembly and structure of energy-storage devices present limitations in terms of weight, size, and rigidity.

Conductive polymers, organic semiconductors, and amorphous silicon have emerged as potential solutions for creating flexible electronics. Nevertheless, a substrate is still necessary to support the conductive material. Commonly used options consist of metal foils, plastics, and glass. As environmental concerns grow, the focus has shifted towards finding more sustainable and reliable alternatives [5,6]. Paper made of cellulose fibers has emerged as a potential substrate for carrying electrodes in several fields where costs, flexibility, and eco-friendliness are important [6,7]. Cellulose is known for being the most abundant biopolymer on earth [8]. Its environmental compatibility, easy handling, and affordability make it an attractive option for different purposes [9]. Most paper is made primarily of cellulose fibers, which do not conduct electricity and must be modified for applications in flexible electronics. Two approaches can be implemented to overcome this limitation. The first approach consists of adding conductive fillers, such as carbonaceous materials [10,11], or conductive polymers (polypyrrole, polyaniline [12,13]) in the papermaking stock so that the formed paper is conductive. Another methodology to impart conductivity is applying a conductive coating on the paper surface [9,14,15,16,17].

In papermaking, aqueous coatings are water-based suspensions comprised of a binder, pigment or filler, and additives, which endow the product with specific characteristics [18]. The binder is important in conductive coatings because it must link the conductive particles together while allowing them to adhere to the substrate. Additionally, the binder should be stable under the operating conditions for the intended application [19]. Different binders and conductive particles have been used to develop this type of coating. Carbon-based materials such as carbon nanotubes (CNTs), single-wall carbon nanotubes (SWCNTs), graphite, graphene, carbon black (CB), activated carbon, and carbon nano-fibers (CNFs) have become promising candidates in this field due to their conductivity, flexibility, eco-friendliness, and possibility of obtaining them from different types of biomasses [20,21].

Given the availability of these environmentally friendly options, several researchers have aimed to formulate conductive coatings implementing carbonaceous components. The work reported by Kossyrev [22] was focused on the development of a carbon black coating for thin electrodes using poly(tetrafluoroethylene) (PTFE) as a binder and isopropyl alcohol as a dispersing agent. Bonnefoi et al. [23] developed electrodes using activated carbon, PTEF, and carboxymethyl cellulose (CMC) as binders. The mixture was spread out on the current collector, and the results indicated that electrodes bound with both PTFE and CMC exhibited improved mechanical properties compared to those that only incorporated CMC. Additionally, using PTFE/CMC mixtures allowed for higher activated carbon content in the electrode, which led to lower resistivity values. Buqa et al. [24] conducted a comprehensive study, testing styrene butadiene rubber (SBR) and various types of modified cellulose (sodium carboxymethyl cellulose, ethyl cellulose, and methyl cellulose) as binders for graphite/silicon electrodes. The study found that styrene butadiene rubber (SBR), sodium carboxymethyl cellulose, or both combined had a similar bonding ability as conventional poly(vinylidene fluoride) (PVDF). Zhu et al. [25] compared the performance of different binders, Nafion^®^ (perfluorosulfonic acid–PTFE copolymer), PTFE, and PVDF, in carbon-based electrodes. It was found that electrodes using PTFE outperformed those using the other two binders.

However, even though progress has been made in conductive coatings comprised of carbonaceous materials, there are few publications where coated paper is used as the substrate for applications in energy storage devices. Liangbing et al. [26] developed conductive coated paper that exhibited sheet resistance as low as 10 Ω/sq. This formulation incorporated single-walled carbon nanotubes (CNTs) with sodium dodecylbenzenesulfonate (SDBS) surfactant. Kordás et al. [27] presented a methodology in which functionalized multi-walled carbon nanotubes (MWCNTs) were sonicated until a homogenous dispersion was achieved. This ink was then printed on paper, reaching a sheet resistance of approximately 40 kΩ/sq.

Tang et al. [28] successfully developed graphite-based coatings that were directly applied onto the surface of low-grade paper made of recycled fibers. The coating formulation comprised graphite suspensions, sodium carboxymethyl cellulose (CMC) and different styrene butadiene (SB) latex concentrations. Results demonstrated that the surface coating technique significantly increased the conductivity of the paper, achieving values ranging from 67 to 276 S/m. Finally, Kumar et al. [16] developed water-based coating formulations, which consisted of nanographite and carbon black as conductive materials. These environmentally friendly coatings included nanocellulose binder as a sustainable alternative to PTFE and latex. The experimental findings indicated that coatings with a mass per unit area of 15 g per square meter exhibited remarkable electrical properties, characterized by surface resistances ranging from 1 to 2 Ω.

However, in the previous studies, the environmental friendliness was compromised by the binders and solvents incorporated. Therefore, in this study, carbon black was used as the conductive coating pigment. Latex (styrene butadiene) and sodium carboxymethyl cellulose (CMC) were examined as binders with the objective of eliminating the need for fluorinated compounds. Additionally, a novel approach is proposed to measure the adhesion of the coating using a mechanical/optical technique. This methodology allows us to determine the strength of the formulation and its integrity when coated on paper.

## 2. Materials and Methods

### 2.1. Conductive Coating

A medium particle-sized, unpelletized carbon black (SR511, iodine adsorption of 44 g iodine/Kg CB, dibutyl phthalate (DBP) absorbed of 127.6 m^3^/100 Kg CB) from Tokai Carbons (Fort Worth, TX, USA) was used. Additional materials included poly(tetrafluoroethylene) (PTFE) as a 60% dispersion in water from Sigma Aldrich St. Louis, MO, USA, ACS grade isopropyl alcohol (Fisher Scientific, Hampton, NH, USA), styrene butadiene (SB) latex as a 55% dispersion in water (Rovene 4100, Mallard Creek, Charlotte, NC, USA) and sodium carboxymethyl cellulose (CMC) in powder with MW ~250,000 and 0.7 degree of substitution (Sigma Aldrich, St. Louis, MO, USA). Deionized water was used as specified.

Several procedures and materials were tested to evaluate different sequences and proportions when incorporating the components. The aim was to determine the formulation with the best dispersion, coating integrity, and electrical conductivity. The concentrations are shown in Table 1. Initially, the coating composition described by Kossyrev [22] was followed in formulation number one, using PTFE and SB latex separately.

Formulations number two through four were tested at different ratios of carbon black/binder. In the case of formulations five through ten, the alcohol concentration was reduced to 70%, 55%, and 40%, respectively, utilizing PTFE and latex separately as binders. Formulations number eleven to sixteen followed the same approach in terms of the alcohol content, but CMC was used as a binder. This methodical variation in composition allowed for a comprehensive analysis of the effects of different binder types and alcohol concentrations on the formulations’ properties.

Formulations 17, 18, and 19 (cf. Table 2) did not include isopropyl alcohol in the liquid medium. The objective was to obtain a more environmentally friendly combination that could be easily incorporated into traditional paper coating systems. Thus, carbon black was added into CMC solutions (0.5, 1, and 2 wt%) following the concentrations shown in Table 2. The resulting mixture was probe-sonicated at room temperature with a Branson Digital sonifier 450 (Emerson Electric Co., St. Louis, MO, USA) for three minutes at 70% amplitude. Additional details regarding the formulations can be found in the Supplementary Materials.

### 2.2. Paper Substrate

Commercial paper with the properties shown in Table 3 was used as substrate for the coating formulations.

The wettability of the sheet was estimated through water contact angle measurements using a Surface Electro-Optics (SEO) contact angle analyzer Phoenix 300 (Surface Electro-Optics Co., San Diego, CA, USA). The paper sample was attached to the sample holder, ensuring the cross-section was clearly visible. Then, a small drop of deionized water was placed on the paper’s surface, and a photo of the instant at which the drop touched the surface was recorded. The contact angle was calculated using the FTA32 software (First Ten Angstroms Inc., Newark, CA, USA, Version 2.1 Build 378). Paper characterization was complemented with scanning electron microscopy (SEM) images using a JCM-6000PLUS-Jeol microscope (JEOL Ltd., Peabody, MA, USA). The basis weight (grams per square meter) was determined according to TAPPI T 410 using the Mettler Toledo (Columbus, OH, USA) analytical balance (PB303-S max = 310 g, min = 0.02, d = 1 mg). The thickness (caliper) was measured following TAPPI T411 using a micrometer (L&W 051, ABB, Zurich, Switzerland). The air permeability was quantified following the TAPPI T 460 Gurley method (indirect measurement of porosity), using the TMI Densometer (Newcastle, DE, USA), recording the number of seconds it took for 100 mL of air to pass through the sheet at 1.2 kPa. Finally, the roughness measurement was performed on both sides of the paper sample, following TAPPI T 555 (Parker print surf method), using an L&W PPS tester (ABB, Zurich, Switzerland).

### 2.3. Coating Technique

The coating formulations were applied using a rod coating technique. The paper substrate was placed on a flat, leveled base and clipped on one edge. An excess of the formulation was applied to the top side of the sheet near the clip. The formulation was immediately drawn to the bottom of the paper, using a Mayer rod N° 6 (16″ length, ½″ diameter), making sure to keep a constant rate without applying additional force beyond the weight of the rod. Subsequently, the coated substrate was left to dry at room temperature, clamping the corners of the paper. This preventive measure addressed potential edge curling induced by moisture present in the coating formulation and swelling of paper fibers.

### 2.4. Coating Evaluation

The coating formulations were evaluated using different tests. Scanning electron microscopy (SEM) images were taken using a JCM-6000PLUS-Jeol (JEOL Ltd., Peabody, MA, USA) microscope and a field-emission scanning electron microscope (FESEM VERIOS 450L, Thermo Fisher Scientific, Waltham, MA, USA). Due to the conductive property of the formulations developed, samples did not require gold coating. For cross-section images, cutting each coated sheet with a one-use cutting blade was necessary. The water contact angle of the coated paper was determined using the technique described in the previous section.

**Figure 1:**
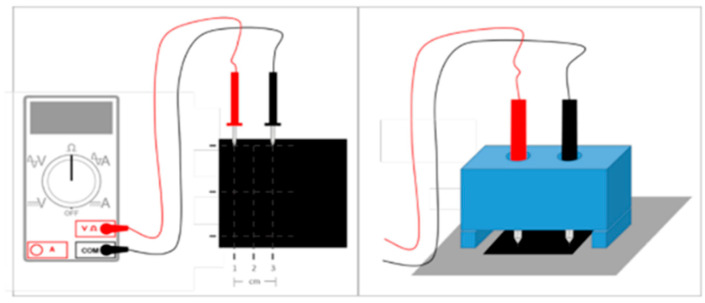
Coating electrical resistance measurements were performed using the arrangement shown to ensure probe position and angle (no pressure added).

Electrical resistance measurements of the coated paper were conducted using the setup illustrated in Figure 1. For these measurements, the probes of a digital multimeter (P37772, CEN-TECH, Calabasas, CA, USA) were positioned at distances of 1, 2, and 3 cm apart, oriented perpendicular to the sample surface. Care was taken to apply only minimal pressure to the probes to prevent any damage to the coating. Prior to measurement, the coated paper substrates were conditioned for 24 h at a temperature of 23.0 ± 1.0 °C and relative humidity of 50.0 ± 2.0%. Electrical resistance readings were then taken in triplicate at various locations on the paper surface to ensure accuracy and reproducibility.

**Figure 2:**
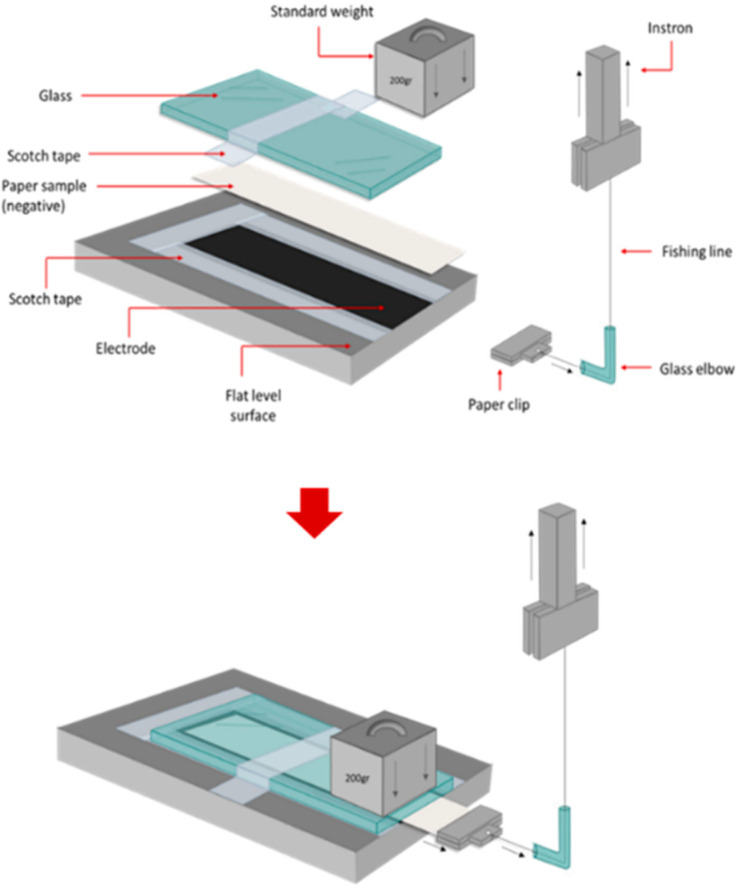
Setup developed to determine coating integrity by paper-coating interface friction analysis.

The coating integrity was evaluated through a measurement technique developed specifically for these materials. The basis of this methodology was to drag a clean white sheet of paper across the surface of the coated substrate with a known normal force and rate of motion. An optical technique known as Estimated Residual Ink Content (ERIC) [29] was used to determine the amount of carbon black coating removed during the sliding. The higher value for the ERIC measurement indicated a more fragile surface, i.e., the carbon black was more easily rubbed from the coated surface. In this test, the coated paper (electrode) was fixed to a flat-level base. An uncoated paper sample (denoted as negative) and a glass slide (7.5 cm × 5 cm) were placed on top of the coated substrate, as shown in Figure 2. The standard mass of 200 g was positioned on top of the glass slide to apply a normal force. Next, a low-stretch/low-friction fine thread was attached to the clean paper sample using a clip. The line was passed through a glass elbow, which was secured in place. The end of the line was then connected to an Instron test frame and dragged at a rate of 125 mm/s. This action pulled the clean sheet out from between the coated paper and the glass slide. TAPPI’s method T 567 was used to measure the Estimated Residual Ink Content (ERIC) value on the side of the negative substrate in contact with the electrode (cf. Figure 2). A ColourTouch (CTX1062) spectrometer from Technidyne Corporation (Industrial Physics, Newcastle, DE, USA) was used to obtain the absorption coefficient of the paper sample containing ink and the ink self-absorption coefficient at a wavelength of 950 nm. The area measured for each sample was 28 ± 3 mm in diameter.

The Estimated Residual Ink Content (ERIC) is the ratio between these two values, expressed unitless or in ppm. This technique has been shown to provide an effective quantification for the residual ink concentration according to the following equation [30]:

(1) $$\mathrm{ERIC}=\left(\frac{k_{\mathrm{sheet}}}{k_{\mathrm{ink}}}\right)\times 10^{6}$$

where

k_sheet_: absorption coefficient of the sheet sample at 950 nm;
k_ink_: absorption coefficient of the ink at 950 nm.

## 3. Results and Discussion

### 3.1. Coating Formulation Stability

**Figure 3:**
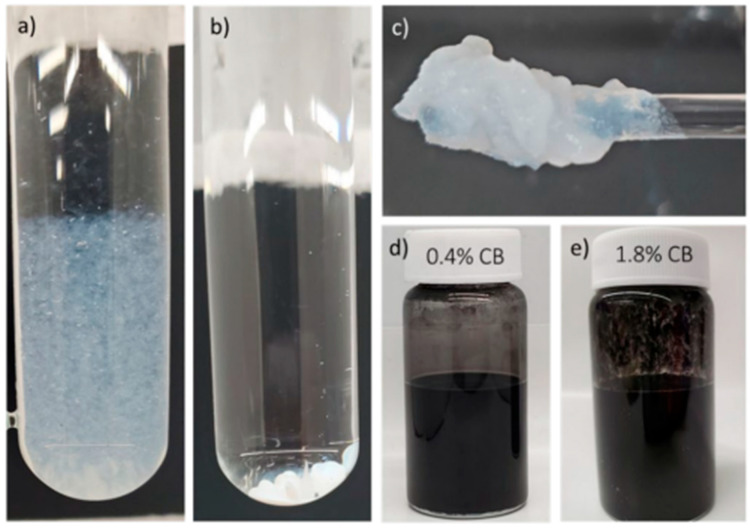
Interaction on different binders with alcohol. (**a**) A mixture of PTFE (polytetrafluoroethylene) and isopropyl alcohol resulted in a gel formation at the bottom of the test tube. (**b**) Precipitation that occurred when using latex as a binder. (**c**) The interaction between PTFE and alcohol produced a gel-like substance. (**d**,**e**) Dispersions at 0.4% and 1.8% of carbon black content.

Isopropyl alcohol was used to disperse carbon black particles, which are hydrophobic and exhibit poor wettability. These characteristics make it difficult to prepare stable dispersions, as they tend to form agglomerates via hydrophobic effects [31,32]. However, it was found that the interaction between the binders and the alcohol generated unwanted interactions, creating a gel for PTFE, CMC, and the precipitation of latex (Figure 3). This behavior was unfavorable for obtaining a homogeneous formulation. Thus, the binders were diluted with DI water prior to mixing with the carbon black–alcohol dispersion in order to overcome the components’ interaction issues. The carbon black dispersion improved at higher alcohol concentrations, and the ratio between isopropyl alcohol/water was crucial. However, an increased alcohol content led to less stable final formulations, which exhibited phase separation over time.

**Figure 4:**
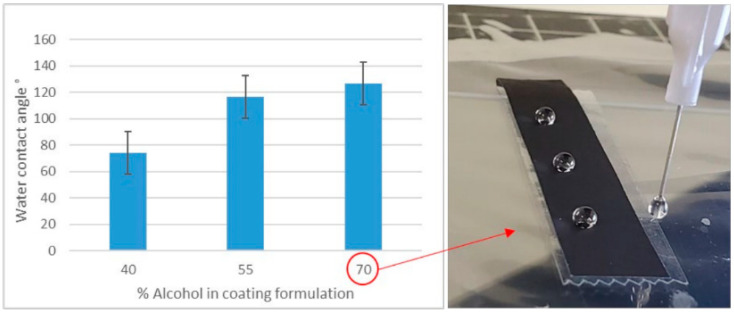
Water contact angle as a function of alcohol concentration in conductive coatings utilizing latex as a binder (formulations 8–10). Increasing alcohol concentration resulted in progressively higher contact angles, with the highest value observed at 70% alcohol concentration.

**Figure 5:**
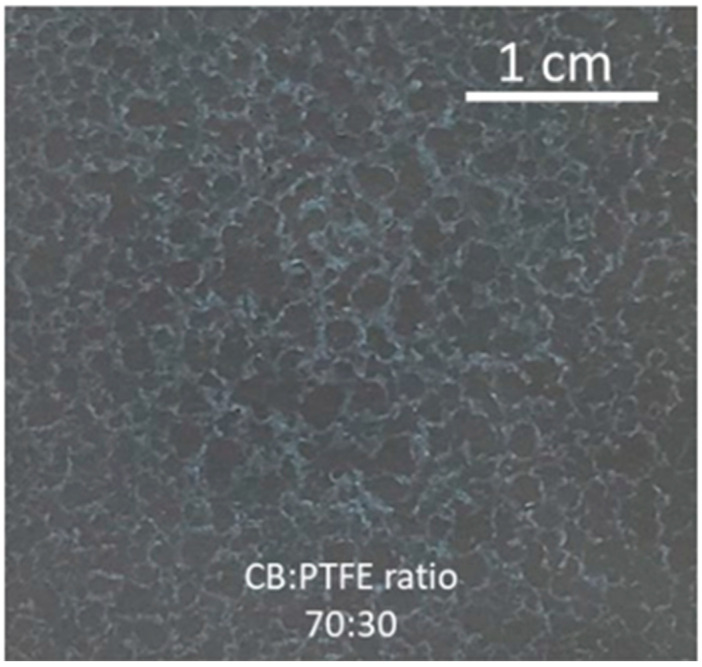
Coated paper using PTFE as binder (formulation number three).

It was found that the type of binder influenced the dispersion stability. Coating formulations were visually observed for uniformity during the working time from making the formulation until coating. The low-stability formulation showed coagulation and separation before coating could be completed. PTFE was found to separate faster when compared to latex or CMC. Additionally, PTFE formulations resulted in a coating with a visually consistent distribution and coverage on the substrate paper. However, the coating exhibited poor adhesion and was easily removed upon light surface rubbing. The reduction in alcohol concentration was observed to reduce coating removal, suggesting increased adhesion between the coating and the paper surface. Furthermore, a reduction in hydrophobicity of the coated substrate was observed, and diminished electrical resistance accompanied this change in the surface energy characteristics. Nevertheless, the drying time increased, and the coating distribution was less homogeneous due to the formation of wrinkles, which may have been due to the swelling of paper fibers. These wrinkles caused surface irregularities, leading to uneven coating coverage. The incorporation of latex as a binder led to better adhesion and more homogeneous application than when using PTFE. These coatings showed increased hydrophobicity, as indicated by higher water contact angles in samples coated with formulations eight to ten (see Figure 4). These observations collectively suggest that the alcohol concentration plays a crucial role in the surface properties and functional performance of latex-based conductive coatings, with higher solvent concentrations leading to more pronounced water-repellent behavior. Although these formulations exhibited better stability, part of the components formed agglomerations that settled to the bottom of the coating container over time. Additionally, increments in binder concentration (formulations number two through four) led to poor coating homogeneity. Figure 5 shows the irregularity of the coating when formulation number three was applied to the paper substrate.

**Figure 6:**
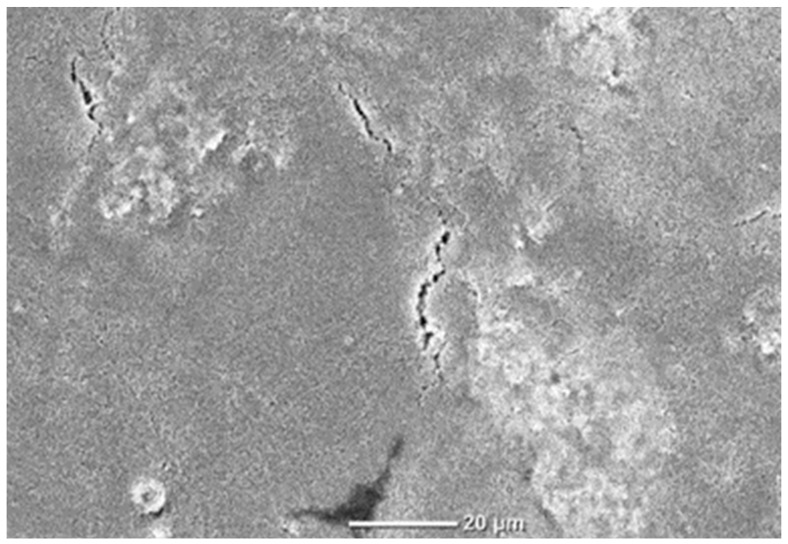
Scanning electron microscopy (SEM) depicting the surface topography of the paper substrate coated with formulation number 18.

Sodium carboxymethyl cellulose was tested as an environmentally friendly binder. These formulations showed better stability, visual consistency, and coating homogeneity than when PTFE or latex was used. (cf. Figure 6). Formulations seventeen to nineteen incorporated CMC as a dispersant and binder. It was found that the best conditions corresponded to formulation number eighteen (1% CMC) in terms of dispersion and consistency when applied with the Mayer rod. As mentioned before, due to the tendency of carbon black particles to form agglomerates, CMC’s anionic carboxymethyl groups impart negative surface charges to conductive particles, counteracting their inherent agglomeration tendencies through electrostatic repulsion [33]. However, formulation seventeen (0.5% CMC) produced an overly viscous mixture, likely due to insufficient dispersion of conductive particles, rendering it incompatible with the rod-coating method. Conversely, formulation nineteen (2% CMC) exhibited poor homogeneity and a rubber-like texture, indicating inadequate coating properties despite higher CMC content.

### 3.2. Effects of Formulation on Resistance and Coating Integrity

Coating formulations with an increased proportion of binding agents (formulations two through four, Table 4) were expected to exhibit superior adhesion properties when applied to paper substrates. This expectation stems from the dual purpose of the binder: firstly, it enhances the cohesive forces between the particles within the dry-coating formulation, creating a more unified mixture; secondly, it promotes stronger adhesive interactions between the coating and the paper surface [34,35]. Therefore, increasing the binder content was aimed at optimizing both the internal stability of the coating and its ability to form a durable, well-adhered layer on the paper. However, increasing the binder concentration led to higher resistance values when incorporating both SB latex and PTFE. This was reflected in the resistance values obtained for formulations two through four (see Table 4). Additionally, the coating was easily removed from the substrate’s surface. The resistance as measured at various spacing can be found in the Supplementary Materials. Data not reported in Table 4 was due to the poor homogeneity of the coating, which prevented measurement of the property.

The positive correlation observed between binder concentration and electrical resistance aligned with the results presented by Tang et al. [28], where the binder amount was carefully optimized to minimize its negative impact on conductivity, as styrene butadiene (SB) latex is non-conductive. Zhu et al. [25] presented similar results, in which a higher binder content led to a decrease in the specific capacitance of the electrodes due to the hydrophobic and insulating nature of these components, which increased internal resistance.

When PTFE was used as a binder, a favorable balance in coating properties was achieved between 70% to 40% alcohol concentration. It is important to note that at higher alcohol concentrations, resistance readings exhibited less stability, leading to significant fluctuations during the measurement process. When using latex, the lowest resistance was achieved with 40% alcohol (formulation number ten). However, the wet coating led to small bubbles forming, contributing to uneven coverage upon drying. Despite this, resistance readings remained consistent.

**Figure 7:**
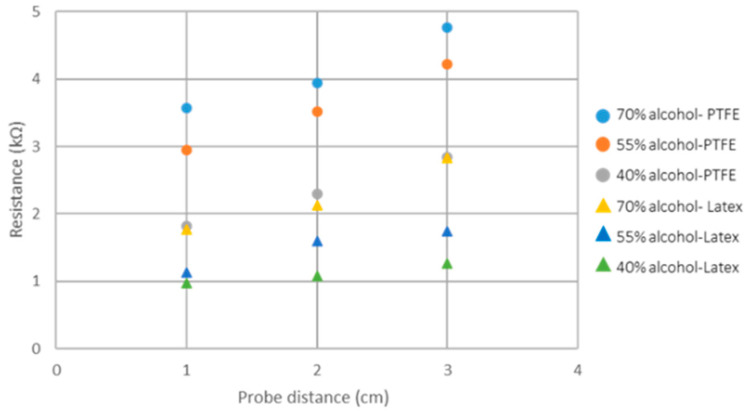
Electrical resistance at different probe distances for conductive cellulose paper when using different alcohol concentrations (PTFE and latex were used separately as binders).

Resistance values of the coated paper using PTFE and SB latex at different alcohol concentrations (formulations five to ten) are shown in Figure 7. The instability of the PTFE formulation at higher alcohol concentrations resulted in greater resistance values, whereas the improved stability of the latex binder gave better resistance values. However, it was determined that lower electrical resistance values were achieved at lower alcohol concentration for both binders. This behavior can be attributed to the rearrangement of solids during extended drying periods caused by increased water content. As the coating dries, the carbon black and binder system is more stable, with higher amounts of water likely leading to a better-formed and more consolidated carbon black structure.

The influence of the conductive particle concentration on resistance and coating adhesion was investigated by evaluating multiple carbon black-to-carboxymethyl cellulose (CB/CMC) ratios. The objective was to determine whether increasing the conductive filler (CB) amount would lead to lower resistance values when using CMC as a binding agent. Formulations with a lower carbon black content (CB/CMC ratio of 4.6) demonstrated better coating integrity, as indicated by lower ERIC numbers. This result was consistent regardless of the alcohol content in the formulation, as shown in Figure 7.

Unexpectedly, it was found that a higher content of the conductive material did not necessarily result in lower resistance values. Formulations eleven to thirteen, with a ratio of CB/CMC of nine, exhibited greater electric resistance (see Table 4) than those with a ratio of four point six (formulations fourteen to sixteen).

**Figure 8:**
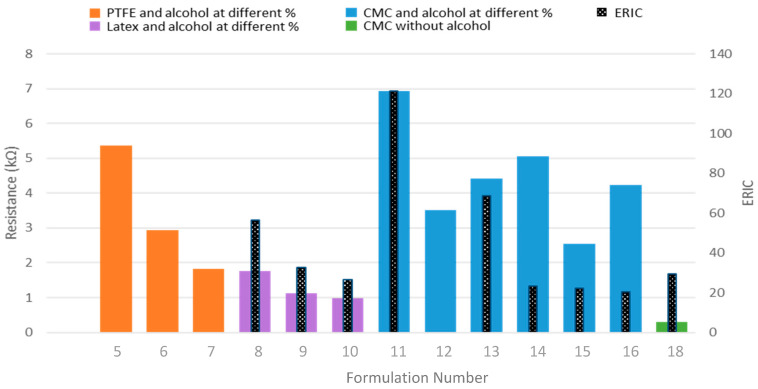
Comparative figure of coating integrity (ERIC) and electrical resistance across diverse coating formulations.

Figure 8 shows the resistance and ERIC values obtained. Formulations five to ten, with an alcohol concentration of 70, 55, and 40%, respectively, exhibited a decrease in resistance at lower alcohol concentrations. Considering coating integrity and resistance value, optimal performance was achieved using latex as a binder with 40% alcohol content (formulation number ten). However, the necessity for a dispersant agent such as isopropyl alcohol compromised the eco-friendliness of the formulation. Using CMC as a binder (formulations eleven to sixteen) resulted in higher coating resistance values than those achieved with PTFE and latex.

This research sought to develop eco-friendly conductive cellulose paper. Therefore, formulations seventeen to nineteen (Table 2) incorporated an aqueous solution of CMC without adding alcohol as a dispersant. It was found that the best results were obtained when this aqueous solution had a concentration in water of 1% CMC, which corresponded to formulation eighteen. When a concentration of 0.5% CMC was used, the resulting formulation was too viscous. Carbon black is known to create agglomerations within the primary particles. The particles are fused by covalent bonds creating aggregates; these aggregates are joined together by van der Walls forces creating agglomerates.

This coating had the best performance in terms of simplicity and non-toxicity, the lowest electrical resistance (0.29 KΩ), and a relatively low ERIC number (better integrity). These results suggest that the CMC-based aqueous formulation offers a promising approach for developing eco-friendly conductive cellulose paper, balancing electrical properties with environmental considerations.

**Figure 9:**
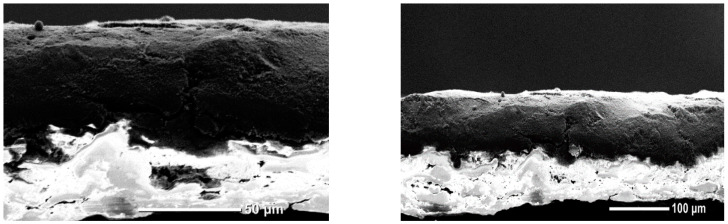
Cross-sectional scanning electron microscopy (SEM) images of conductive cellulose paper coated with formulation eighteen, which incorporated CMC as a dispersant and binder.

SEM cross-section images of the coated substrate (using formulation number eighteen) showed that the coating was able to penetrate the sample voids and cover the fiber surface, instead of forming a continuous separated layer on top of it (see Figure 9). The apparent coating thickness was 51.9 (SD = 6.4) µm. The rapid spreading of the conductive coating with the Mayer rod impeded its thorough permeation. However, other variables need to be considered to evaluate the coating continuity, such as porous structure, roughness, and water-resistant treatments applied to the paper substrate.

## 4. Conclusions

Several methods were used to create a carbon black-based conductive formulation to coat a cellulose paper substrate. The type of binder, dispersant, and concentration were studied. The results showed that formulations using PTFE and latex as binders required isopropyl alcohol as a dispersant. However, PTFE-based coatings were hydrophobic and lacked strong adhesion to the paper, while latex-based coatings demonstrated better adhesion and lower electrical resistance. Increasing the alcohol content led to reduced coating stability, especially for PTFE formulations, which also exhibited higher resistance due to discontinuous coating layers. Additionally, the presence of alcohol altered the hydrophobicity of latex-based coatings. It was found that the formulation composition critically affected electrical performance, with higher concentrations of non-conductive components, such as binders, increasing sheet resistance. Seeking a more environmentally friendly alternative, carboxymethyl cellulose (CMC) was found to be an effective dispersant for carbon black, enabling the elimination of alcohol and the difficulties associated with it. A carbon black-to-CMC ratio of seven yielded the lowest resistance (0.29 kΩ) and good coating integrity among all tested formulations. More importantly, this formulation can be considered more environmentally friendly since it eliminates the need for a volatile organic compound (isopropyl alcohol).

## Figures and Tables

**Table 1:** Coating compositions using alcohol as solvent.

98% Solvent (Composition)		2% Solids (Composition)				
Condition Evaluated	Formulation	Binder	Isopropyl Alcohol ACS Grade (wt%)	DI Water (wt%)	Carbon Black (wt%)	Binder (wt%)
Kossyrev [22] formulation	1	PTFE and latex (separately)	99.9	0.10	90	10
CB/binder ratio	2	PTFE and	97.7	0.3	80	20
	3	latex	97.6	0.4	70	30
	4	(separately)	97.3	0.7	50	50
Alcohol concentration	5		70	30	90	10
	6	PTFE	55	45	90	10
	7		40	60	90	10
Alcohol concentration	8	Latex	70	30	90	10
	9		55	45	90	10
	10		40	60	90	10
Alcohol concentration	11		42	58	90	10
	12	CMC	33	67	90	10
	13		24	76	90	10
CB/binder ratio (at different alcohol %)	14		42	58	82	18
	15	CMC	33	67	82	18
	16		24	76	82	18

**Table 2:** Coating compositions without alcohol.

Formulation	DI Water (wt%)	Solids (wt%)	Solids Composition	
			CB (wt%)	CMC (wt%)
17	96	4	88	13
18	93	7	88	13
19	86	14	88	13

**Table 3:** Paper characterization results.

Property	Basis Weight (g/m^2^)	Caliper (µm)	Density (g/cm^3^)	Contact Angle (°)	Air Permeability (s/100mL)	Roughness (µm)
Average	44.94	64	705.55	91.31	68.70	5.61
Standard Deviation	0.45	0.87	1.01 × 10^−5^	2.98	3.02	0.44

**Table 4:** Electrical resistance and ERIC values for different carbon black formulations.

Condition Evaluated	Formulation	Binder	Resistance (kΩ)		Standard Deviation		ERIC		Standard Deviation	
Kossyrev [22] formulation	1	PTFE and latex (separately)	-		-		-		-	
CB/binder ratio	2	PTFE and	PTFE	Latex	PTFE	Latex	PTFE	Latex	PTFE	Latex
		latex	10.38	6.24	1.78	4.81	-	-	-	-
	3	(separately)	9.77	3.81	4.26	1.46	-	-	-	-
	4		5.07	1.74	0.32	0.28	-	-	-	-
Alcohol concentration	5		5.37		0.2		-		-	
	6	PTFE	2.94		0.6		-		-	
	7		1.82		0.25		-		-	
Alcohol concentration	8	Latex	1.77		0.37		56.31		15.66	
	9		1.13		0.17		32.62		8.38	
	10		0.97		0.03		26.44		3.42	
Alcohol concentration	11		6.94		1.41		121.19		22.94	
	12	CMC	3.52		0.73		-		-	
	13		4.42		0.70		68.85		21.12	
CB/binder ratio (at different alcohol %)	14		5.06		0.60		23.12		3.24	
	15	CMC	2.55		0.28		22.33		3.4	
	16		4.24		0.61		20.22		4.66	
Using CMC as binder and dispersant	18	CMC	0.29		0.56		29.49		2.13	

## Data Availability

The original contributions presented in this study are included in the article. Further inquiries can be directed to the corresponding author.

## Supplementary Materials

The following supporting information can be downloaded at: https://www.mdpi.com/article/10.3390/ma18122708/s1, Supplementary File: Supplementary Information Millian et al.
