# Supplementary material for: Coating Formulations Based on Carbon Black: An Alternative to Develop Environmentally Friendly Conductive Cellulose Paper
Source: Materials (Basel). 2025 Jun 9;18(12):2708. doi: 10.3390/ma18122708 (PMC12194713; doi:10.3390/ma18122708)
Supplement: Supplementary file 1 [file materials-18-02708-s001.zip › materials-3635826-supplementary.pdf]

Supplementary Information for: "Coating formulations based on carbon black: an alternative to develop environmentally friendly conductive cellulose paper."

Adriana Millan[a], Anny Morales[b], Richard Venditti[c], Joel Pawlak[d],

[a] Department of Forest Biomaterials, College of Natural Resources, North Carolina State University, Raleigh, North Carolina 27695-8005, United States.
[b] Department of Forest Biomaterials, College of Natural Resources, North Carolina State University, Raleigh, North Carolina 27695-8005, United States. **Email**: acmorale@ncsu.edu
[c] Department of Forest Biomaterials, College of Natural Resources, North Carolina State University, Raleigh, North Carolina 27695-8005, United States. **Email**: richardv@ncsu.edu
[d] Department of Forest Biomaterials, College of Natural Resources, North Carolina State University, Raleigh, North Carolina 27695-8005, United States. **Email**: jjpawlak@ncsu.edu
* Correspondence: **Author:**
[d] Department of Forest Biomaterials, College of Natural Resources, North Carolina State University, Raleigh, North Carolina 27695-8005, United States. **Email**: jjpawlak@ncsu.edu

Formulations tested:

**CMC**					
	**98 % solvent**		**2 % solids**		
Concentration CMC base solution	**Isopropyl alcohol ACS grade %**	**DI Water %**	**Carbon black %**	**CMC %**	**CMC solution %**
0.50%	58	40	90	10	0.5
	42	58	90	10	0.4
	33	67	90	10	0.3
	24	76	90	10	0.3
1%	60	40	82	18	1
	42	58	82	18	0.7
	33	67	82	18	0.6
	24	76	82	18	0.5
2%	80	20	80	20	2
	56	44	80	20	0.9
	44	56	80	20	0.7
	32	68	80	20	0.6

**LATEX**	
**98 % solvent**	**2 % solids**

	Isopropyl alcohol ACS grade %	DI Water %	Carbon black %	Latex %
Decreasing latex content	70	30	95	5
	55	45	95	5
	40	60	95	5
Varying alcohol %	70	30	90	10
	55	45	90	10
	40	60	90	10

	PTFE			
	98 % solvent		2 % solids	
	Isopropyl alcohol ACS grade %	DI Water %	Carbon black %	PTFE %
Original	99.9	0.1	90	10
Varying CB/binder ratio	97.7	0.3	80	20
	97.6	0.4	70	30
	97.3	0.7	50	50
Varying alcohol %	70	30	90	10
	55	45	90	10
	40	60	90	10

Electrical resistance of PTFE as a binder:

Nomenclature	1cm	2cm	3cm
**ACB-WP_70/30**	3.78	4.5	5.13
	3.38	3.24	3.97
	3.54	4.09	5.19
	**3.57**	**3.94**	**4.76**
**St. dev.**	***0.20***	***0.64***	***0.69***
**ACB-WP_55/45**	3.55	3.95	4.93
	2.93	3.59	3.98
	2.35	3.02	3.75
	**2.94**	**3.52**	**4.22**
**St. dev.**	***0.60***	***0.47***	***0.63***
**ACB-WP_40/60**	1.9	2.43	2.9
	1.54	2.02	2.23
	2.02	2.43	3.38
	**1.82**	**2.29**	**2.84**
**st dev**	**0.25**	**0.24**	**0.58**

Electrical resistance of latex as a binder:

Nomenclature	1cm	2cm	3cm
**ACB-WL_70/30**	**1.58**	**2.15**	**2.92**
	1.54	1.74	2.48
	2.2	2.47	3.1
	**1.77**	**2.12**	**2.83**
**St. dev.**	***0.37***	***0.37***	***0.32***
**ACB-WL_55/45**	1.23	1.91	1.97
	0.93	1.42	1.55
	1.23	1.45	1.71

	**1.13**	**1.59**	**1.74**
**St. dev.**	***0.17***	***0.27***	***0.21***
**ACB-WL_40/60**	0.95	1.07	1.18
	0.96	0.98	1.05
	1.01	1.18	1.54
	**0.97**	**1.08**	**1.26**
**St. dev.**	***0.03***	***0.10***	***0.25***

Coating thickness measure for formulation number 18:

Coat thickness (µm)	Coat thickness (µm)	Coat thickness (µm)
48.327	38.682	52.832
50.373	38.682	61.8
47.205	41.992	52.864
52.799	50.009	53.792
49.815	38.679	44.342
58.583	45.755	45.283
61.009	53.302	50.952
56.721	43.399	50.481
55.971	48.585	54.717
58.769	51.415	
56.716	47.644	
52.985	58.019	
49.819	62.264	
56.903	58.019	
59.52	58.021	
60.823	49.528	
Average	51.91	
